# Real-Time Microbiology Laboratory Surveillance System to Detect Abnormal Events and Emerging Infections, Marseille, France

**DOI:** 10.3201/eid2108.141419

**Published:** 2015-08

**Authors:** Cédric Abat, Hervé Chaudet, Philippe Colson, Jean-Marc Rolain, Didier Raoult

**Affiliations:** Aix-Marseille Université, Marseille, France (C. Abat, P. Colson, J.-M. Rolain, D. Raoult);; Sciences Economiques et Sociales de la Santé et Traimtement de l’Information Médicale (SESSTIM), Marseille (H. Chaudet)

**Keywords:** microbiology, antimicrobial resistance, public health, data collection, epidemiology, bacteria, early warning, syndromic surveillance

## Abstract

Infectious diseases are a major threat to humanity, and accurate surveillance is essential. We describe how to implement a laboratory data–based surveillance system in a clinical microbiology laboratory. Two historical Microsoft Excel databases were implemented. The data were then sorted and used to execute the following 2 surveillance systems in Excel: the Bacterial real-time Laboratory-based Surveillance System (BALYSES) for monitoring the number of patients infected with bacterial species isolated at least once in our laboratory during the study periodl and the Marseille Antibiotic Resistance Surveillance System (MARSS), which surveys the primary β-lactam resistance phenotypes for 15 selected bacterial species. The first historical database contained 174,853 identifications of bacteria, and the second contained 12,062 results of antibiotic susceptibility testing. From May 21, 2013, through June 4, 2014, BALYSES and MARSS enabled the detection of 52 abnormal events for 24 bacterial species, leading to 19 official reports. This system is currently being refined and improved.

## Abstract 2

We implemented a laboratory data–based syndromic surveillance system that can be rapidly applied and used as a first tool.

Although infectious diseases were declared under control and considered to be a past public health problem during the second half of the 20th century (*1*), these diseases, including those that are well-known, emerging, and reemerging, remain a major threat to humanity. Indeed, infectious pathogens possess an amazing common capacity to emerge and spread in unpredictable ways before they are detected by public health institutions (*2*). Infectious diseases have a substantial effect on both global human demographics (they are the second leading cause of death in humans worldwide, accounting for ≈15 million deaths) (*3*) and the economy (*4*), which has led the public health community to reconsider them as a real threat. This alarming observation has led public health authorities to try to improve infectious disease surveillance.

One of these strategies, known as traditional public health surveillance of infectious diseases, has been to use clinical case reports from sentinel laboratories or laboratory networks and direct reports of positive results from clinical laboratories to survey the presence of microbial agents known to be dangers to health in a precise population (*5*). Some examples of surveillance systems implemented by using this strategy are the National Tuberculosis Surveillance System in the United States (*6*), the surveillance system of the Netherlands Reference Laboratory for Bacterial Meningitis (*7*) and the European Gonococcal Antimicrobial Surveillance Programme (*8*).

Another strategy, known as syndromic surveillance, consists of developing real-time surveillance systems capable of detecting abnormal epidemiologic events, not on the basis of infectious disease diagnosis data, but rather on the basis of nonspecific health indicators, such as absenteeism, chief complaints, and prescription drug sales (*5*,*9*). Such surveillance systems can be implemented nationally, such as the Emergency Department Syndromic Surveillance System in England (*10*) or the National Retail Data Monitor in the United States (*11*), and regionally, such as the Emergency Department Syndromic Surveillance in Canada (*12*) or the European Antimicrobial Resistance Surveillance Network in Europe (*13*), or the systems can be administered by laboratories with large quantities of data and the financial and human resources to apply the information.

On the basis of our experience at the Assistance Publique–Hôpitaux de Marseille (AP-HM), we describe all the steps necessary for implementing a laboratory data–based syndromic surveillance system in a laboratory. Because of its simplicity, we believe that it can be rapidly applied and used as a first surveillance tool in well-established laboratories. We also show the advantages and limits of this surveillance system.

## Materials and Methods

### Study Setting

Marseille is the second-most populous French city (estimated population 850,726 persons in 2010). All data analyzed in this article came from the 4 university hospitals of Marseille (North, South, Conception, and Timone hospitals). Cumulatively, these hospitals represent ≈3,700 beds, including ≈1,500 beds for the Timone Hospital, ≈600 beds for the North Hospital, ≈700 beds for the Conception Hospital, and ≈900 beds for the South Hospital. The AP-HM clinical microbiology laboratory is located at Timone Hospital; the laboratory performed ≈145,000 serologic tests and ≈200,000 PCRs and cultures of microorganisms from 220,000 samples in 2012 (*14*). This amount of data allowed us to implement our own laboratory-data–based syndromic surveillance system.

### Organization of Surveillance Activity on Tools of AP-HM

**Figure 1:**
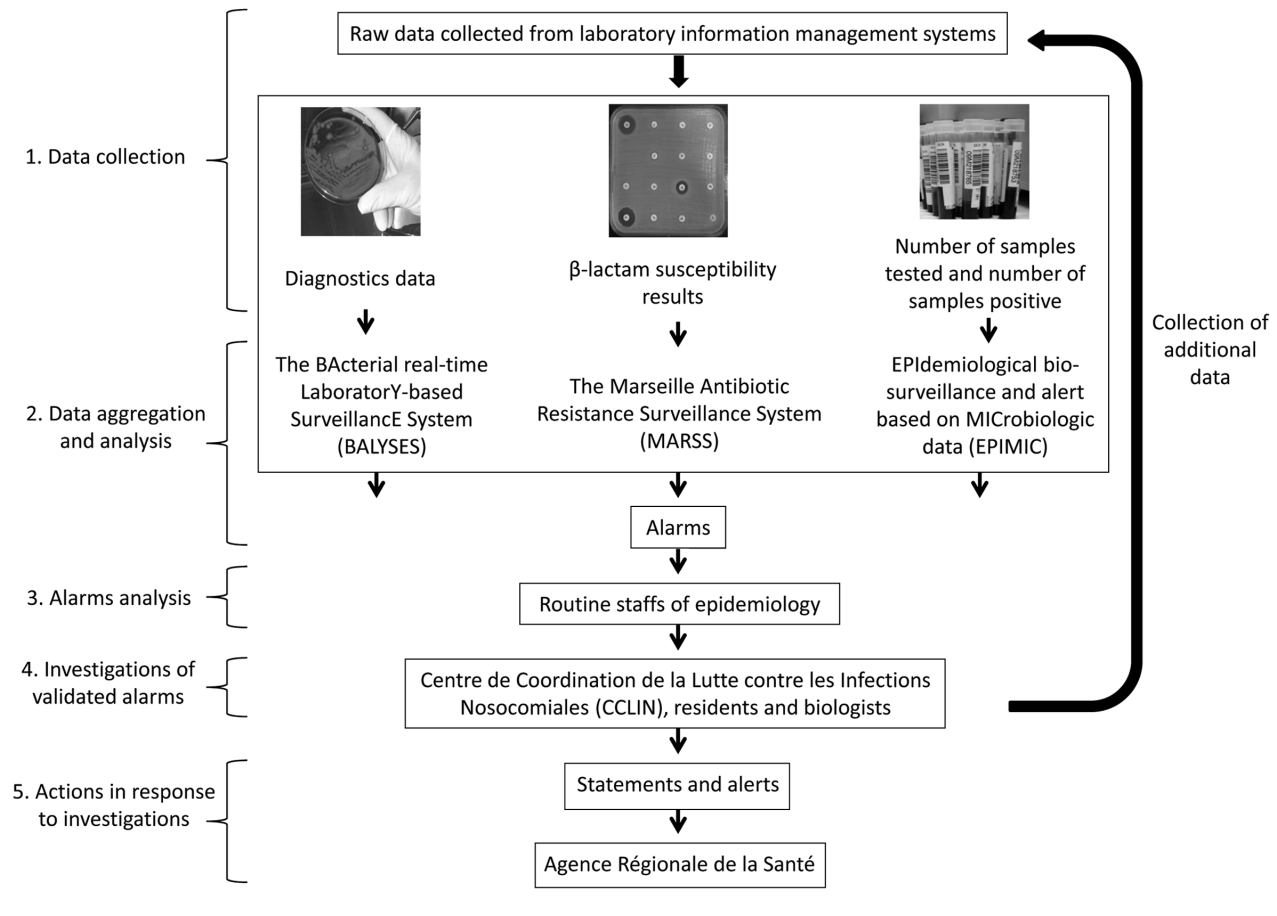
Workflow of real-time surveillance systems used by Institut Hospitalo–Universitaire Méditérranée Infection, Assistance Publique-Hôpitaux de Marseille, Marseille, France.

The AP-HM laboratory–based surveillance consists of 3 following syndromic surveillance tools founded on Excel software (Mircosoft Corp., Redmond, WA, USA): 1 previously described system called EPIMIC (EPIdemiological biosurveillance and alert based on MICrobiologic data) (*15*,*16*), 1 surveillance system implemented for the surveillance of bacterial antibiotic resistance (MARSS, Marseille Antibiotic Resistance Surveillance System), and BALYSES (BActerial real-time LaboratorY-based SurveillancE System), which was developed for the surveillance of the number of patients infected by each bacteria species identified at least once in our laboratory. Our surveillance systems are defined as syndromic surveillance systems because no surveillance data are specifically collected for their use. The flow of information needed for each of the 3 surveillance systems is summarized in Figure 1. However, only BALYSES and MARSS are further described.

All of the data routinely used for the 2 surveillance systems are manually collected from the Timone Hospital laboratory information management systems and processed by using Microsoft Excel software (2007 version). Data are then entered in the 2 surveillance systems according to their nature. The 2 systems automatically compare the entered data with their specific thresholds. Alarms are emitted by the systems if the entered values exceed thresholds. The emitted alarms are analyzed weekly during a specific thematic epidemiology meeting with laboratory staff. If alarms are validated, further investigations are immediately conducted by biologists, clinicians, and medical residents. After the alarm is signaled, our institution’s team in charge of nosocomial infections, called the Centre de Coordination de la Lutte contre les Infections Nosocomiales, initiates an investigation. Finally, if these investigations reveal that the alarm events were real epidemiologic events (thereafter called true alarms), official reports can be sent to an official regional public health institution, the Agence Régionale de la Santé (ARS).

### Laboratory Data–Based Syndromic Surveillance System

#### BALYSES

**Figure 2:**
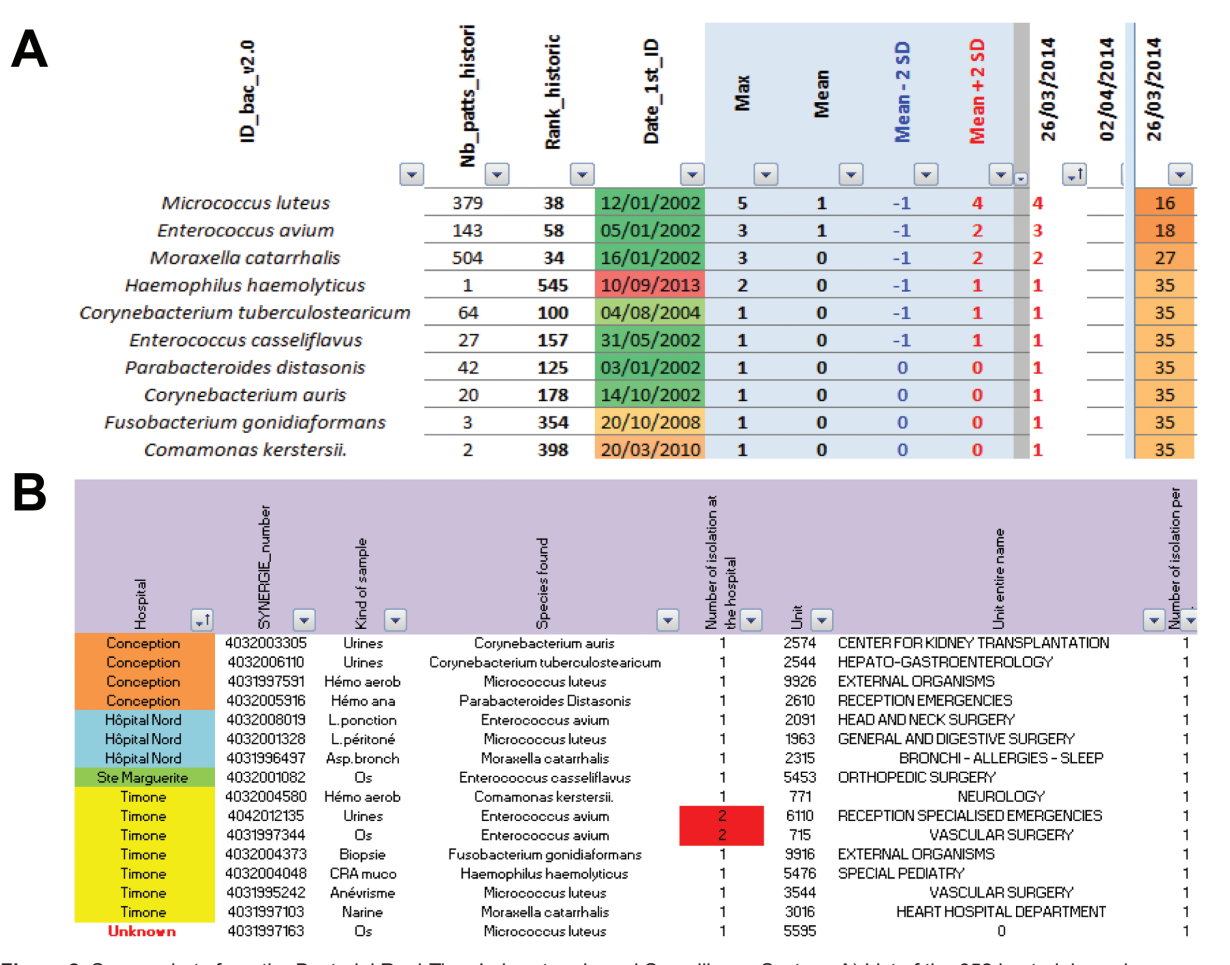
Screen shots from the Bacterial Real-Time Laboratory-based Surveillance System. A) List of the 652 bacterial species followed by the Bacterial Real-time Surveillance System and all of the contained information. B) Interface summarizing information from the alarms. ID_bac_v2.0, all the bacterial species followed by the surveillance system; Nb_patts_histori, the historical number of patients infected by the bacterium; Rank_historic, the historical rank of a precise bacterium under surveillance; Date_1st_ID, the date of first identification of the bacterium.

The BALYSES surveillance system was implemented and has been routinely used since January 2013. The first version of BALYSES was implemented to automatically compare the weekly number of samples positive for each bacterial species identified at least once at our institution with the mean historical weekly values ± 2 SDs (Table 1). In October 2013, BALYSES was improved to survey the weekly number of patients infected by each bacterial species (Figure 2; Table 1). Then, if alarms are emitted that indicate an abnormal increase in the number of isolations of a specific bacterial species, an additional Microsoft Excel interface is used to show more details, including the hospitals and units in which the patients received care, the types of samples from which the bacterial species were isolated, and the patients’ identification numbers. BALYSES also automatically classifies the bacterial species from most to least abundant, according to the weekly number of infected patients, and calculates their weekly rank. It finally calculates the maximum number of patients infected by each of the bacterial species monitored, indicates the date of first isolation of the bacterial species at AP-HM, and identifies the historical rank (on the basis of the historical number of patients infected) among the other bacterial species.

**Table 1:** Validated alarms emitted by BALYSES and investigations from May 21, 2013 through June 4, 2014, Marseille, France*

Pathogen	Alarm date	Abnormal event observed	Weekly mean no. samples or patients, ± 2 SDs, rounded values	Investigation	Result	Intervention
*Klebsiella oxytoca*	2013 May 28	Abnormal increase in no. positive samples, with 11 positive samples from 5 patients	10	Additional investigations did not identify any links between infected patients	False alarm	No intervention
*Raoultella ornithinolytica*	2013 Jun 4	Abnormal increase in no. positive samples, with 2 positive samples from 2 patients	2	The 2 patients were hospitalized in t same intensive care unit of same hospital	True alarm	Report sent to ARS
*Morganella morganii*	2013 Jun 18	Abnormal increase in no. positive samples, with 16 positive samples from 3 patients	12	Additional investigations did not identify any links between infected patients	False alarm	No intervention
*Aeromonas hydrophila*	2013 Jul 7	Abnormal increase in no. positive samples, with 1 positive sample from 1 patient	1	Additional investigations confirmed that patient was infected by the bacterium (leeches used to cure him were infected)	True alarm	Patient was cured with antibiotics, and report was sent to ARS
*Enterococcus faecium*	2013 Jul 16	Abnormal increase in no. positive samples, with 16 positive samples	14	Additional investigations did not identify any links between infected patients	False alarm	No intervention
*Enterobacter aerogenes*	2013 Jul 16	Abnormal increase in no. positive samples, with 13 positive samples	11	Additional investigations did not identify any links between infected patients	False alarm	No intervention
*E. aerogenes*	2013 Jul 30	Abnormal increase in no. positive samples, with 18 positive samples from 7 patients	13	Additional investigations did not identify any links between infected patients	False alarm	No intervention
*Klebsiella oxytoca*	2013 Jul 30	Abnormal increase in no. positive samples, with 13 positive samples from 4 patients	10	Additional investigations did not identify any links between infected patients	False alarm	No intervention
*Enterobacter cloacae*	2013 Sep 3	Abnormal increase in no. positive samples, with 31 positive samples from 15 patients	25	Additional investigations did not identify any links between the infected patients	False alarm	No intervention
*Staphylococcus capitis*	2013 Oct 1	Abnormal increase in no. patients infected, with 7 patients infected	7	Additional investigations did not identify any links between infected patients, and no clones were identified by MALDI-TOF spectra analysis	False alarm	No intervention
*Staphylococcus hominis*	/2013 Oct 1	Abnormal increase in no. patients infected, with 13 patients infected	13	Additional investigations did not identify any links between the infected patients and no clones were identified by MALDI-TOF spectra analysis	False alarm	No intervention
*E. cloacae*	2013 Oct 8	Abnormal increase in no. patients infected, with 25 patients infected	25	True nosocomial transmission of the pathogen was identified between some infected patients	True alarm	Report sent to ARS
*E. aerogenes*	2013 Oct 29	Abnormal increase in no. patients infected, with 13 patients infected	10	True nosocomial transmission of the pathogen was identified between some infected patients	True alarm	Report sent to ARS
*Staphylococcus gallolyticus*	2013 Nov 5	Abnormal increase in no. patients infected, with 4 patients infected	3	Additional investigations did not identify any links between the infected patients and no clones were identified by MALDI-TOF spectra analysis	True alarm	No intervention
*Gardnerella vaginalis*	2013 Dec 3	Abnormal increase in no. patients infected, with 25 patients infected	25	Additional investigations did not identify any links between infected patients	True alarm	No intervention
*Haemophilus parahaemolyticus*	2013 Dec 17	Abnormal increase in no. patients infected, with 3 patients infected	2	True nosocomial transmission of the pathogen between infected patients	True alarm	Retrospective analysis of patients revealed that the bacterium was isolated from children with cystic fibrosis (whole genome sequencing of the strains is ongoing)
*Staphylococcus saprophyticus*	2014 Jan 7	Abnormal increase in no. patients infected, with 5 patients infected	5	Additional investigations did not identify any links between infected patients, and no clones were identified by MALDI-TOF spectra analysis	False alarm	No intervention
*Escherichia coli*	/2014 Jan 21	Abnormal increase in no. patients infected, with 206 patients infected	191	Additional investigations did not identify any links between infected patients, and no clones were identified by MALDI-TOF spectra analysis	True alarm	No explanations were found; first results indicate that most infections come from women with community acquired urinary-tract infections
*M. morganii*	2014 Feb 4	Abnormal increase in no. patients infected, with 13 patients infected	10	Additional investigations did not identify any links between infected patients, and no clones were identified by MALDI-TOF spectra analysis	False alarm	No intervention
*Moraxella catarrhalis*	2014 Feb 11	Abnormal increase in no. patients infected with 3 patients infected	2	Additional investigations did not identify any links between infected patients, and no clones were identified by MALDI-TOF spectra analysis	False alarm	No intervention
*Aeromonas hydrophila*	2014 Mar 11	Abnormal increase in no. positive samples, with 1 positive sample from 1 patient	1	Additional investigations confirmed that patient was infected by the bacterium (leeches used to cure him were infected)	True alarm	Patient was cured with antibiotic drugs; report sent to ARS

*BALYSES, BActerial real-time LaboratorY-based SurveillancE System; MALDI-TOF, matrix-assisted laser desorption ionization time of flight; ARS, Agence Régionale de Santé.

#### MARSS

**Figure 3:**
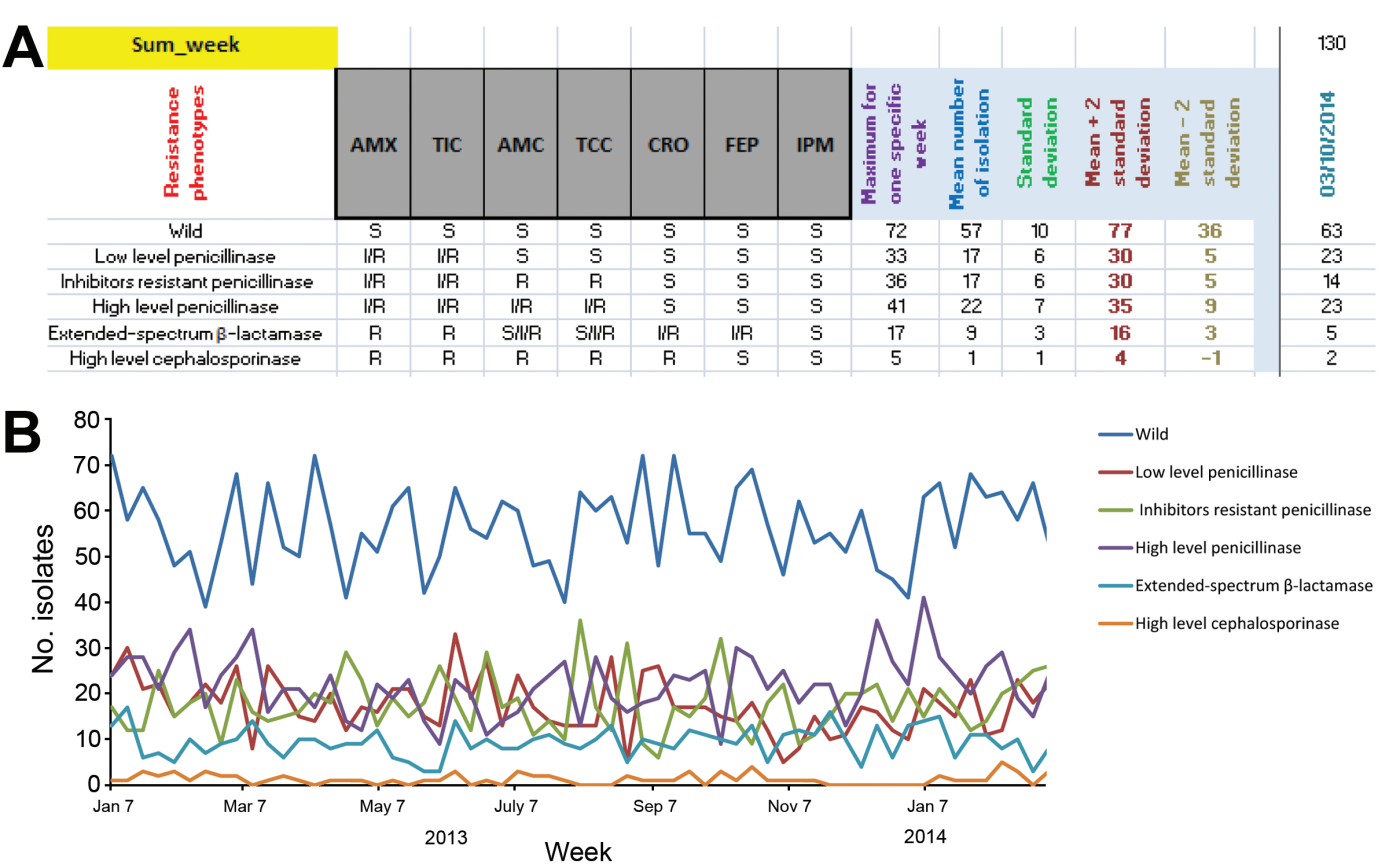
Marseille Antibiotic Resistance Surveillance System (MARSS) interface for *Escherichia coli*. A) Screen shot showing list of most of the β-lactam antibiotic resistance profiles coded for *E. coli* in MARSS. B) Example of graph created by using MARSS showing the evolution of the antibiotic resistance of *E. coli*.

The MARSS surveillance program has been used since April 2013. Fifteen bacterial species are monitored by MARSS, including *Escherichia coli, Klebsiella pneumoniae*, *K. oxytoca*, *Proteus mirabilis*, *Enterobacter cloacae*, *Enterobacter aerogenes, Morganella morganii*, *Serratia marcescens, Pseudomonas aeruginosa*, *Acinetobacter baumannii*, *Streptococcus agalactiae*, *Enterococcus faecalis, Enterococcus faecium*, *Staphylococcus aureus,* and *S. epidermidis*. MARSS automatically compares the weekly number of isolates exhibiting a given β-lactam resistance phenotype to the mean value ± 2 SDs for the historical number of strains harboring this phenotype (Figure 3). Alarms are emitted when this threshold is exceeded. In parallel, MARSS emits alarms for key phenotypes to allow for their rapid identification and verification (Table 2, 3).

**Table 2:** Summary of the normal phenotypes registered in MARSS*

Bacterial species	Resistance phenotypes	β-lactam antibiotics										
		AMX	TIC	AMC	TCC	TZP	FOX	OXA	CRO	FEP	CAZ	IPM
*Escherichia coli*												
	Wild-type	S	S	S	S				S	S		S
	Low-level penicillinase	I/R	I/R	S	S				S	S		S
	Inhibitor-resistant penicillinase	I/R	I/R	R	R				S	S		S
	High-level penicillinase	I/R	I/R	I/R	I/R				S	S		S
	ESBL	R	R	S/I/R	S/I/R				I/R	I/R		S
	High-level cephalosporinase	R	R	R	R				R	S		S
*Klebsiella pneumoniae*	Wild-type			S		S			S	S		S
	ESBL			I/R		I/R			I/R	I/R		S
	High-level cephalosporinase			I/R		I/R			I/R	S		S
	ESBL-TZP-sensible			I/R		S			I/R	I/R		S
*Proteus mirabilis*	Wild	S	S	S	S				S	S		S
	Low-level penicillinase	I/R	I/R	S	S				S	S		S
	Inhibitor-resistant penicillinase	R	R	R	R				S	S		S
	High-level penicillinase	R	R	I/R	I/R				S	S		S
	ESBL	R	R	I/R	I/R				I/R	I/R		S
	High-level cephalosporinase	R	R	R	R				R	S		S
*Klebsiella oxytoca*	Wild-type			S		S				S		S
	ESBL			I/R		I/R				I/R		S
	High-level penicillinase			I/R		S/I/R				S		S
	Low-level penicillinase			S		R				S		S
	ESBL–TZP-sensible			I/R		S				I/R		S
*Enterobacter aerogenes*	Wild-type				S	S			S	S		S
	Inhibitors-resistant penicillinase				R	R			S	S		S
	ESBL				S/I/R	I/R			I/R	I/R		S
	High-level cephalosporinase				I/R	I/R			I/R	S		S
*Morganella morganii*	Wild-type				S	S			S	S		S
	Inhibitor-resistant penicillinase				R	R			S	S		S
	ESBL				S/I/R	I/R			I/R	I/R		S
	High-level cephalosporinase				I/R	I/R			I/R	S		S
*Serratia marcescens*	Wild-type				S	S			S	S		S
	Inhibitor-resistant penicillinase				R	R			S	S		S
	ESBL				S/I/R	I/R			I/R	I/R		S
	High-level cephalosporinase				I/R	I/R			I/R	S		S
*Enterobacter cloacae*	Wild-type				S	S			S	S		S
	Inhibitor-resistant penicillinase				R	R			S	S		S
	ESBL				S/I/R	I/R			I/R	I/R		S
	High-level cephalosporinase				I/R	I/R			I/R	S		S
*Pseudomonas aeruginosa*	Wild-type		S		S	S				S	S	S
	Penicillinase		R		R/I/S	I/S				S	S	S
	High-level penicillinase		I/R		I/R	I/R				S	S	S
	ESBL		I/R		I/R	I/R				I/R	I/R	S
	Selective permeability to imipenem		S		S	S				S	S	R
	Penicillinase, loss of D2 porine		R		R	S				S	S	R
*Acinetobacter baumannii*	Wild-type		S		S						S	S
	Penicillinase		R		R/I/S						S	S
	ESBL		I/R		I/R						I/R	S
*Streptococcus agalactiae*	Wild							S	S			
	Oxacillin-resistant							I/R	S			
*Enterococcus faecalis*	Wild-type	S										
*E. faecium*	Wild-type	I/R										
*Staphylococcus aureus*	Wild-type						S					
	Methicillin-resistant						I/R					
*S. epidermidis*	Wild-type						S					
	Methicillin-resistant						I/R					

*MARSS, Marseille Antibiotic Resistance Surveillance System; AMC, amoxicillin; TIC, ticarcillin; AMC, amoxicillin/clavulanic acid; TIC, ticarcillin/clavulanic acid; TZP, piperacillin/tazobactam; FOX, cefoxitin; OXA, oxacillin; CRO, ceftriaxone; FEP, cefepime; CAZ, ceftazidime; IMP, imipenem; S, susceptible; I, intermediate; R, resistant; ESBL, extended-spectrum β-lactamase.

**Table 3:** Summary of the alarm phenotypes defined in MARSS*

Bacteria species	Alarm triggering key phenotypes
*Escherichia coli, Proteus mirabilis*	Carbapenem resistance
*Klebsiella pneumoniae*	Carbapenem resistance
*Klebsiella oxytoca*	Carbapenem resistance
*Enterobacter aerogenes*, *Morganella morganii*, *Serratia marcescens,* *Enterobacter cloacae*	Carbapenem resistance
*Pseudomonas aeruginosa*	Carbapenem resistance
*Acinetobacter* spp.	Carbapenem and colistin resistance
*Streptococcus agalactiae*	Ceftriaxone resistance
*Enterococcus faecalis*	Amoxicillin resistance
*Enterococcus faecium*	Amoxicillin susceptible
*Staphylococcus aureus*	Vancomycin resistance

*MARSS, Marseille Antibiotic Resistance Surveillance System.

### Historical Databases

The detection of abnormal events necessitates the calculation of expected references, previously called historical thresholds. To define the expected references, 2 historical databases were built by using data extracted from the laboratory information management systems of the 4 university hospitals of Marseille. The first historical database consisted of all of the bacterial identifications obtained from January 2002 to December 2013 (excluding December 2002, data unavailable), including those described in a previous work (*17*), and a second database consisted of most antimicrobial resistance profiles obtained from October 2012 through March 2013. These data were then processed with Microsoft Excel software (2007 version) and sorted. The first database was then sorted, and only samples from which bacterial species were properly identified were conserved. Then, the duplicates for patient and bacterial species were removed. The second database was sorted into different Microsoft Excel spreadsheets for the most frequently isolated bacterial species. Duplicates occurring within the same week were then removed on the basis of the same methods.

## Results

### Databases and Surveillance Systems

The first version of the 11-year historical BALYSES database contained 161,374 bacterial identifications corresponding to 568 different bacterial species. The 10 most numerous bacterial species were *E. coli* (37,560 patients), *S. aureus* (23,562 patients), *S. epidermidis* (11,091 patients), *P. aeruginosa* (9,113 patients), *K. pneumoniae* (7,576 patients), *E. faecalis* (7,403 patients), *S. agalactiae* (4,473 patients), *E. cloacae* (4,453 patients), *P. mirabilis* (4,415 patients), and *Haemophilus influenzae* (2,424 patients). The 2013 updates increased the number of bacterial identifications to 174,853 and the number of monitored bacterial species to 611 (43 new bacterial species were added). Among them, 384 bacterial species, defined here as rare bacterial species, were identified <11 times in the 12-year period.

The historical MARSS database included 12,062 antibiograms from October 2012 to March 2013. Here, the 10 most frequently isolated bacterial species were *E. coli* (3,293 strains), *S. aureus* (1,613 strains), *Achromobacter xylosoxidans* (1,478 strains), *S. epidermidis* (822 strains), *E. faecalis* (749 strains), *K. pneumoniae* (729 strains), *P. mirabilis* (455 strains), *S. agalactiae* (322 strains), *E. cloacae* (278 strains), and *Staphylococcus hominis* (153 strains).

### Alarms Validated and Investigated, May 21, 2013– June 4, 2014

**Figure 4:**
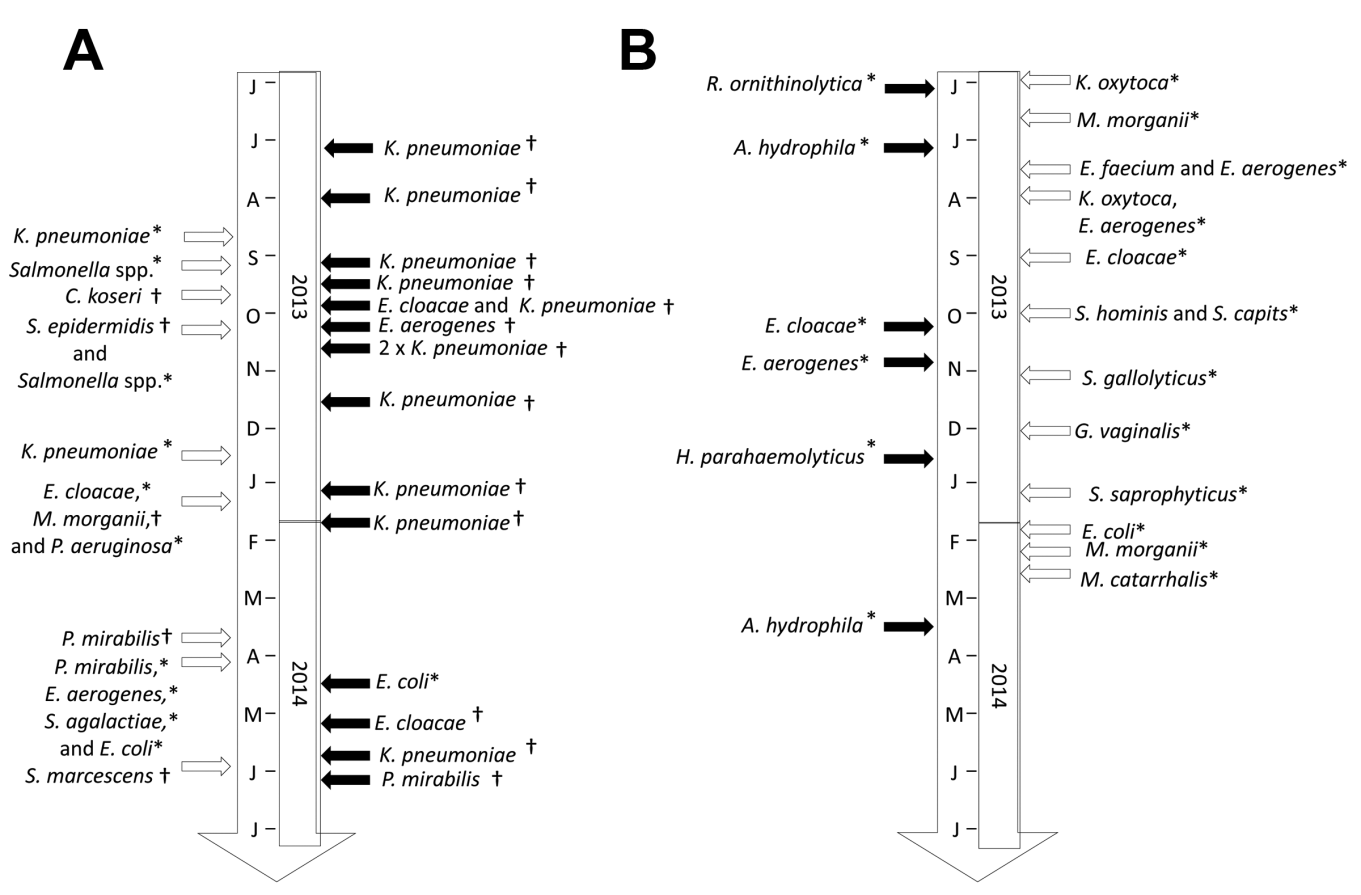
Time chart of the confirmed and unconfirmed events identified by the Marseille Antibiotic Resistance Surveillance System (MARSS) and the Bacterial real-time Laboratory-based Surveillance System (BALYSES). A) List of all the abnormal events (confirmed or not) detected by MARSS. B) List of all the abnormal events (confirmed or not) detected by BALYSES. Open arrows, unconfirmed events; solid arrows, confirmed events; asterisk (*), alarm due to abnormal increases or abnormal isolations; dagger (†), alarm due to strain with abnormal antibiotic susceptibility results.

From May 21, 2013, through June 4, 2014 (55 weeks), BALYSES detected 21 alarms (6 confirmed events and 15 unconfirmed events), corresponding to ≈0.4 alarms per week. These alarms led to 5 official reports to the ARS of the Provence-Alpes-Côte d’Azur (PACA) region, France (Table 1; Figure 4). The positive predictive value for the study period was 0.28. Sixteen bacterial species triggered alarms in this surveillance system. The bacterial species that triggered alarms were *E. aerogenes* (3 alarms), *Aeromonas hydrophila* (2 alarms), *E. cloacae* (2 alarms), *K. oxytoca* (2 alarms), *M. morganii* (2 alarms), *E. coli* (1 alarm), *E. faecium* (1 alarm), *Gardnerella vaginalis* (1 alarm), *Haemophilus parahaemolyticus* (1 alarm), *Moraxella catarrhalis* (1 alarm), *Raoultella ornithinolytica* (1 alarm), *Staphylococcus capitis* (1 alarm), *Staphylococcus gallolyticus* (1 alarm), *Staphylococcus hominis* (1 alarm), and *Staphylococcus saprophyticus* (1 alarm). As an example of the system’s usefulness, BALYSES allowed us to detect a real nosocomial transmission of *R. ornithinolytica* between 2 patients in the intensive care unit at the Timone Hospital on June 4, 2013 (Table 1).

In parallel, MARSS detected 31 alarms (16 confirmed events and 15 unconfirmed events, ≈0.6 alarms/week), which led to 15 official reports to the ARS of the PACA region, France (Table 4; Figure 4). The positive predictive value for the study period was 0.52. Thirteen bacterial species triggered alarms in MARSS. Here, the bacterial species, in order according to the number of alarms triggered, were *K. pneumoniae* (13 alarms), *E. cloacae* (3 alarms), *P. mirabilis* (3 alarms), *E. coli* (2 alarms), *E. aerogenes* (2 alarms), *Salmonella* spp. (2 alarms), *P. aeruginosa* (1 alarms), *Citrobacter koseri* (1 alarm), *M. morganii* (1 alarm), *S. marcescens* (1 alarm), *S. epidermidis* (1 alarm), and *S. agalactiae* (1 alarm). As an example of the system’s usefulness, MARSS allowed us to detect a local outbreak of oxicillinase-48 carbapenemase–producing *K. pneumoniae* from July 2013 to October 2013 (11 patients infected) (unpub. data; Table 4).

**Table 4:** Validated alarms emitted by MARSS and investigations from May 21, 2013, through June 4, 2014, Marseille, France*

Pathogen	Alarm date	Abnormal event observed	Investigation	Result	Intervention
*Klebsiella pneumoniae*	2013 Jul 9	Isolation from 1 patient of *K. pneumoniae* strain that seemed to produce carbapenemases	Additional antibiotic susceptibility testing confirmed that strain was carbapenemase producing	True alarm	Patient isolated and resport sent to ARS
*K. pneumoniae*	2013 Jul 30	Isolation from 1 patient of *K. pneumoniae* strain that seemed to produce carbapenemases	Additional antibiotic susceptibility testing confirmed that strain was carbapenemase producing	True alarm	Patient isolated and resport sent to ARS
*K. pneumoniae*	2013 Aug 20	Abnormal increase in no. strains with ESBLphenotype	Additional investigations led to conclusion that increase was due to a technical problem in MARSS	False alarm	Technical problem was immediately corrected
*K. pneumoniae*	2013 Sep 9	Isolation from 1 patient of *K. pneumoniae* strain that seemed to produce carbapenemases	Additional antibiotic susceptibility testing confirmed that strain was carbapenemase producing	True alarm	Patient isolated and resport sent to ARS
*Salmonella* spp.	2013 Sep 10	3 strains isolated from 3 patients	Additional investigations did not identify any links between infected patients	False alarm	No intervention
*K. pneumoniae*	2013 Sep 10	Isolation from 1 patient of *K. pneumoniae* strain that seemed to produce carbapenemases	Additional antibiotic susceptibility testing confirmed that strain was carbapenemase producing	True alarm	Patient isolated and resport sent to ARS
*Citrobacter koseri*	2013 Sep 17	Various strains isolated with same antibiotic-resistance profile	Additional investigations did not identify any links between infected patients	False alarm	No intervention
*Enterobacter cloacae*	2013 Sep 20	Isolation from 1 patient of *E. cloacae* strain that seemed to produce carbapenemases	Additional antibiotic susceptibility testing confirmed that strain was carbapenemase producing	True alarm	Patient isolated and resport sent to ARS
*K. pneumoniae*	2013 Sep 24	Isolation from 1 patient of *K. pneumoniae* strain that seemed to produce carbapenemases	Additional antibiotic susceptibility testing confirmed that strain was carbapenemase producing	True alarm	Patient isolated and resport sent to ARS
*Enterobacter aerogenes*	2013 Oct 4	Isolation from 1 patient of *E. aerogenes* strain that seemed to produce carbapenemases	Additional antibiotic susceptibility testing confirmed that strain was carbapenemase producing	True alarm	Patient isolated and resport sent to ARS
*Staphylococcus epidermidis*	2013 Oct 8	Abnormal increase in no. *S. epidermidis* strains highly resistant to methicillin	Additional investigations did not identify any links between infected patients, and no clones were identified with MALDI-TOF spectra analysis	False alarm	No intervention
*Salmonella* spp.	2013 Oct 8	Abnormal increase in no. strains isolated from patients hospitalized in same hospital	Additional investigations did not identify any links between infected patients, and no clones were identified by MALDI-TOF spectra analysis	False alarm	No intervention
*K. pneumoniae*	2013 Oct 15	Isolation from 1 patient of *K. pneumoniae* strain that seemed to produce carbapenemases	Additional antibiotic susceptibility testing confirmed that strain was carbapenemase producing	True alarm	Patient isolated and resport sent to ARS
*K. pneumoniae*	2013 Oct 15	Isolation from 1 patient of *K. pneumoniae* strain that seemed to produce carbapenemases	Additional antibiotic susceptibility testing confirmed that strain was carbapenemase producing	True alarm	Patient isolated and resport sent to ARS
*K. pneumoniae*	2013 Nov 22	Isolation from 1 patient of *K. pneumoniae* strain that seemed to produce carbapenemases	Additional antibiotic susceptibility testing confirmed that strain was carbapenemase producing	True alarm	Patient isolated and resport sent to ARS
*K. pneumoniae*	2013 Dec 10	Abnormal increase in no. strains with ESBL resistance phenotype	Additional investigations did not identify any links between infected patients, and no clones were identified by MALDI-TOF spectra analysis	False alarm	No intervention
*K. pneumoniae*	2013 Jan 3	Isolation from 1 patient of *K. pneumoniae* strain that seemed to produce carbapenemases	Additional antibiotic susceptibility testing confirmed that strain was carbapenemase producing	True alarm	Patient isolated and report sent to ARS
*E. cloacae*	2014 Jan 7	Abnormal increase in no. *E. cloacae* strains with wild resistance phenotype isolated for week of 2013 Nov 25p[	Additional investigations did not identify any links between infected patients, and no clones were identified by MALDI-TOF spectra analysis	False alarm	No intervention
*Morganella morganii*	2014 Jan 7	Abnormal increase in no. *M. morganii* strains isolated with inhibitor-resistant penicillinase phenotype	Additional investigation allowed us to conclude it was a wild phenotype strain	False alarm	No intervention
*Pseudomonas aeruginosa*	2014 Jan 7	Abnormal increase in no. *P. aeruginosa* strains with wild-type resistance phenotype isolated for 28/10/2013 week	Additional investigations did not identify any links between infected patients, and no clones were identified by MALDI-TOF spectra analysis	False alarm	No intervention
*K. pneumoniae*	2014 Jan 21	Isolation from 1 patient of *K. pneumoniae* strain that seemed to produce carbapenemases	Additional antibiotic susceptibility testing confirmed that strain was carbapenemase producing	True alarm	Patient isolated and resport sent to ARS
*Proteus mirabilis*	2014 Jan 25	Isolation from 1 patient of *P. mirabilis* strain that seemed to produce carbapenemases	Additional antibiotic susceptibility testing did not confirm that strain was carbapenemase producing	False alarm	No intervention
*Enterobacter aerogenes*	2014 Apr 8	Abnormal increase in no. strains with high level cephalosporinase resistance phenotype	Additional investigations did not identify any links between infected patients, and no clones were identified with MALDI-TOF spectra analysis	False alarm	No intervention
*Escherichia coli*	2014 Apr 8	Abnormal increase in no. strains with inhibitor-resistant penicillinase phenotype	Additional investigations did not identify any links between infected patients, and no clones were identified with MALDI-TOF spectra analysis	False alarm	No intervention
*P. mirabilis*	2014 Apr 8	Abnormal increase in no. strains with wild phenotype	Additional investigations did not identify infected patients, and no clones were identified with MALDI-TOF spectra analysis	False alarm	No intervention
*Streptococcus agalactiae*	2014 Apr 8	Abnormal increase in no. strains with methicillin-resistant phenotype	Additional investigations did not identify any links between infected patients, and no clones were identified with MALDI-TOF spectra analysis	False alarm	No intervention
*E. coli*	2014 Apr 8	Abnormal increase in no. strains with inhibitor-resistant phenotype	Additional MALDI-TOF spectra analysis did not identify clones	True alarm	No explanations were found. First results from ongoing investigations show that most infections come from community-acquired urinary tract infections in women
*E. cloacae*	2014 Apr 8	Isolation from 1 patient of *E. cloacae* strain that seemed to produce carbapenemases	Additional antibiotic susceptibility testing confirmed that strain was carbapenemase producing	True alarm	Patient isolated and resport sent to ARS
*K. pneumoniae*	2014 May 21	Isolation from 1 patient of *K. pneumoniae* strain that seemed to produce carbapenemases	Additional antibiotic susceptibility testing confirmed that strain was carbapenemase producing	True alarm	Patient isolated and resport sent to ARS
*Serratia marcescens*	2014 Jun 3	Isolation from 1 patient of *S. marcescens* strain that seemed to produce carbapenemases	Additional antibiotic susceptibility testing did not confirm that strain was carbapenemase producing	False alarm	No intervention
*P. mirabilis*	2014 Jun 4	Isolation from 1 patient of *P. mirabilis* strain that seemed to produce carbapenemases	Additional antibiotic susceptibility testing confirmed that strain was carbapenemase producing	True alarm	Patient isolated and resport sent to ARS

*MARSS, Marseille Antibiotic Resistance Surveillance System; ESBL*,* extended spectrum β-lactamase*;*ARS, MALDI-TOF, matrix-assisted laser desorption ionization time of flight; ARS, Agence Régionale de Santé.

For clarification, not all of the true alarms led to official reports because we did not identify the reasons why these abnormal increases occurred (Table 1, 4). Nevertheless, investigations are ongoing to try to elucidate these phenomena.

## Discussion

### Analysis of 2 Real-Time Laboratory-Based Surveillance Systems

Implementing surveillance systems on the basis of data that were not specifically collected for surveillance is one of the advantage of our systems. Indeed, these types of systems, syndromic surveillance systems, are well suited in places and situations in which surveillance tools are urgently needed (*18*). In our situation, this approach allowed us to rapidly implement the system and quickly detect abnormal events related to bacterial infections occurring in our institution (19 official reports) (Table 1, 4; Figure 4).

The fact that all of the emitted alarms are systematically validated during epidemiologic meetings with microbiologists (Figure 1) is also a strength of this laboratory surveillance system. Thus, the system enables rapid verification and filtering of false alarms to ensure that the official reports sent to the regional health authorities (ARS) are correct. This facilitates a rapid public health response to counter possible epidemics. As an example, EPIMIC, our third surveillance system not described here (Figure 1) (*15*,*16*), allowed us to detect a nosocomial outbreak of the hypervirulent *Clostridium difficile* ribotype O27 that started in March 2013 (*19*). As we continue to fight this major public health problem, a list of recommended containment measures, such as systematic isolation of infected patients in special care units or systematic screening of patients at risk, is being published and transmitted to our institutional and regional health care providers.

Our 2 surveillance systems have been implemented by using Microsoft Excel software. This strategy makes the systems easy to handle and allows rapid modifications and improvements without the need for in-depth computer skills. These advantages may not be the case for fully designed website surveillance systems such as the Swiss Antibiotic Resistance Surveillance database (*20*) or the Real-Time Outbreak and Disease Surveillance (RODS) (*21*). These aspects are key factors for the optimal long-term use at the hospital level because surveillance systems can be considered complex socio-technical systems with the objective of assisting users during abnormal epidemic events (*22*).

The implementation of our 2 surveillance systems required 1 full-time PhD student for 4 months and a computer with standard configuration equipped with Microsoft Office version 2003 or 2007. In France, the national research agency requires that the minimum salary of a PhD student is 33,000€ per year. Considering that the average price for a basic computer equipped with Microsoft Office is ≈500€ and that the PhD student’s salary for the 4 months was 11,000€, plus the administrative and management costs, the total consolidated cost of these surveillance systems was ≈13,800€ (US $17,000).

The use of our own microbiology laboratory data ensures the availability and the completeness of the data. These problems are frequently mentioned when surveillance systems collect data from various health care institutions. For example, the designers of the German Surveillance System of Antibiotic Use and Bacterial Resistance encountered problems comparing antibiogram data between participating intensive care units. Indeed, in Germany, laboratories did not apply 1 standard to determine antibiotic-resistance profiles of the bacterial species (*23*). Moreover, the increasing number of intensive care units joining the surveillance system may effect the comparability of collected data because recently added intensive care units may use different antibiotic drugs, thus leading to different antimicrobial resistance profiles (*24*). Poor quality data were also observed in the emergency department syndromic surveillance system in New York, primarily because of the lack of human resources (*25*).

However, our surveillance systems have 2 main limitations. The first limitation is the statistical analysis used for the detection of abnormal events. As described before, our surveillance systems compared entered data with the historical means ± 2 SDs. For our purposes, this tool was simple to develop and was used effectively to detect abnormal events. However, these statistics do not consider seasonal variations in pathogen isolation, especially for rare bacterial species. To address this problem, Enki et al. improved the detection algorithms according to the frequency of isolation of the 3,303 pathogens included in the 20-year LabBase surveillance database recovered from the UK Health Protection Agency (*26*). They discovered that although all of these organisms varied greatly in their isolation frequency, most of them could be surveyed by using quasi-Poisson or negative binomial models for which the variance is proportional to the mean. In MARSS, the use of moving averages in our kinetic graphs or of cumulative sum control charts, as has been done in RODS (http://openrods.sourceforge.net/), could also be effective improvements for the detection of abnormal events.

The second limitation was that all of the data in our system were manually collected and entered into the surveillance system. This aspect can introduce bias into our data analysis. For example, we have already observed false alarms after shifts in data collection because of national holidays or because of the lack of human resources, which is a problem also observed in other surveillance systems, such as the emergency department syndromic surveillance system in New York (*25*). To address these issues, simple solutions can be developed, such as implementing and using informatic tools for automatic collection and processing of the collected data. This solution was implemented by the designers of ASTER, the French military decision-supported surveillance system (*22*).

With knowledge of the previously mentioned weaknesses, we are currently working to improve our 2 surveillance systems. Thus, a surveillance platform that will merge all of the surveillance activities and will contain stronger statistical tools for the surveillance of abnormal events is under development. This platform will help us survey abnormal events by using all of the clinical microbiology data available in the laboratory. Moreover, our monitoring activity is expanding to other laboratories in the PACA region. We are implementing a regional laboratory surveillance system that will allow us, on the basis of the clinical microbiology data that are collected every week, to gain a better understanding of the local dissemination of pathogens at the regional level and to survey weekly isolation frequencies. Finally, another surveillance system based on matrix-assisted laser desorption/ionization–time of flight spectra of bacteria is currently under development in our laboratory. A prototype is used weekly in our laboratory to try to detect epidemics, including the possible nosocomial transmission of bacterial clones.
